# Turkish adaptation of the Artificial Intelligence Self-Efficacy Scale (AISES) and its role in prospective teachers’ intentions to use AI in classroom practices

**DOI:** 10.1186/s40359-026-05014-2

**Published:** 2026-06-23

**Authors:** Fatih Aydın, İbrahim Dadandı, Şenel Çıtak, Vildan Saki Aydın, Pakize Urfalı Dadandı

**Affiliations:** 1 Faculty of Education, Department of Educational Sciences, Sivas Cumhuriyet University, Sivas, 58140 Turkey; 2 Faculty of Education, Department of Educational Sciences, Yozgat Bozok University, Yozgat, 66900 Turkey; 3 Faculty of Education, Department of Educational Sciences, Ordu University, Ordu, 52200 Turkey; 4 Faculty of Education, Department of Educational Sciences, Trabzon University, Trabzon, 61300 Turkey; 5 Faculty of Education, Department of Turkish and Social Sciences Education, Yozgat Bozok University, Yozgat, 66900 Turkey; 6 Faculty of Education, Yozgat Bozok University, Erdoğan Akdağ Doğu Kampüsü Atatürk Yolu 7. Km, Yozgat, 66900 Turkey

**Keywords:** AI literacy, AI self-efficacy, Attitudes toward AI, Behavioral intention, Teacher education (UN SDG 4)

## Abstract

**Background:**

With groundbreaking advancements in digital technologies, AI tools have been adopted in everyday life in a short time, including in educational settings. The aim of the present study was to adapt the Artificial Intelligence Self-Efficacy Scale in Turkish and to examine how AI literacy, attitudes, and self-efficacy predict prospective teachers’ intentions to use AI in their future classroom practices.

**Methods:**

In this regard, a total of 610 prospective teachers from the Faculties of Education at three public universities in Turkey were surveyed using a battery of questionnaires to evaluate AI literacy, AI self-efficacy, attitudes towards AI, and behavioral intention to use AI in teaching.

**Results:**

Confirmatory factor and reliability analyses indicated that the Turkish version of the AISES demonstrated good psychometric properties. Additionally, the serial mediation model demonstrated that prospective teachers’ levels of AI literacy significantly predicted their self-efficacy, attitudes, and intentions regarding future classroom practices. Moreover, the relationship between AI literacy and behavioral intention is mediated sequentially by AI self-efficacy and attitudes toward AI.

**Conclusions:**

The findings of the present study indicate that enhancing prospective teachers’ AI literacy and self-efficacy can foster positive attitudes and strengthen their intentions to use AI technologies in future educational practices.

**Supplementary Information:**

The online version contains supplementary material available at https://doi.org/10.1186/s40359-026-05014-2.

## Introduction

Technological advancements have significantly contributed to almost every aspect of everyday life over the last century, including educational settings. Tools such as computers, 3D printers, the internet, video and audio content, electronic books, and learning management systems have become commonplace in classrooms, benefiting both teachers and students. Digital technologies enable students to access information anytime and anywhere. They also facilitate the creation and sharing of educational content, thereby allowing them to take a more proactive role in the learning. In addition, they may assist teachers with routine duties such as preparing teaching materials and monitoring student performance [1]. More recently, artificial intelligence (AI) has attracted growing interest from researchers and educators worldwide and has raised a key question: Can AI help create better learning environments?

Growing evidence suggests that integrating AI into education—commonly referred to as AIEd—may enhance students’ learning outcomes by fostering more flexible, personalized, adaptive, and engaging learning environments [2]. AI can also be employed to design more effective learning activities and support technology-enhanced, intelligent classrooms [3]. Both sides of the teacher-learner dynamic benefit: teachers can delegate certain tasks to AI applications, while students receive personalized content, feedback, and guidance tailored to their preferences, abilities, and developmental stages [4, 5]. As Pham and Sampson [6] highlight, if implemented effectively, AIEd can lead to remarkable improvements in educational outcomes. For instance, Bughin and colleagues reported that an AI-powered adaptive learning program at Arizona State University increased pass rates and reduced dropout rates in an arithmetic course [7].

AI holds the potential to improve the learning process in a variety of ways. It may enhance teacher efficiency by supporting both administrative tasks (e.g., grading, scheduling, tracking student progress, detecting plagiarism, and providing feedback) and instructional tasks (e.g., content delivery, lesson planning, personalized curriculum design, and syllabus preparation) [8, 9]. AI tools—often referred to as “cobots” or “intelligent AI tutors” [10, 11]—such as Carnegie Cognitive Tutor, Duolingo, Squirrel AI, and others, are now used to assist students in subjects such as mathematics and English language teaching [12–14]. Many of these tasks are time-consuming and cognitively demanding without AI support. By automating these tasks, AI may give teachers more time for professional development, creative planning, and direct student support [15, 16].

For learners, AI provides continuous access to personalized feedback, allows them to monitor their strengths and weaknesses, and helps tailor their learning paths to meet their unique needs and goals. It can also assist with career planning by providing tailored resources and recommendations [17]. Moreover, AI applications support the development of global classrooms through real-time AI translation, promote lifelong learning, and help cultivate 21st-century skills in students [18]. AI is increasingly recognized for its ability to influence educational policymaking beyond individual classrooms [3]. By analyzing vast datasets, it can identify academic trends, risk factors, and the elements that contribute to student success, thereby providing evidence-based recommendations for improving educational systems. In this regard, the adoption of new AI technologies by educators in educational settings as assistive teaching tools is crucial for transforming their potential benefits into practical educational outcomes. Research has identified several factors that influence teachers’ willingness to embrace AI technologies. Even when teachers have similar levels of technological competence, they may use technology differently in classrooms [6]. Some rely on it for basic tasks, such as delivering presentations, while others use it for more complex purposes, including promoting student interaction, problem-solving, and research and development projects [19]. Various barriers, such as a preference for traditional teaching methods [20], the perceived difficulty and effort required to learn new technologies [21], or a lack of confidence in one’s own skills [22], may discourage teachers from adopting technology. These individual and contextual differences in technology adoption are also relevant for prospective teachers and can be better understood through theoretical models that explain users’ acceptance and use of new technologies.

The technology acceptance model (TAM) posits that many personal, behavioral, and contextual factors influence an individual’s intention to utilize technology [23]. The TAM model identifies key constructs that are related to individuals’ actual use of new technologies through their behavioral intentions [24]. The original model, developed by Davis [25], suggests that actual system usage is shaped by users’ attitudes toward the technology, which in turn are influenced by perceived usefulness and ease of use. The model was subsequently extended to TAM 2, which incorporates additional factors such as subjective norms, job relevance, and prior experience [26]. It was further expanded into TAM 3, which includes individual differences, social influence, facilitating conditions, and system characteristics [27]. More recent efforts to synthesize accumulated insights across these models resulted in the unified theory of acceptance and use of technology (UTAUT) and its extension, UTAUT 2 [28, 29]. Drawing on this framework, the present study examines the factors associated with prospective teachers’ behavioral intentions to use AI applications.

### The current study and the development of the hypotheses

As AI technologies become widespread, research underscores the need to modify teacher education curricula to enhance future teachers’ AI skills and competencies [30]. Therefore, identifying the factors that determine prospective teachers’ behavioral intention to use AI may provide valuable insights for teacher education programs. Behavioral intention is a key component of the UTAUT2 model [29], and recent studies have argued that integrating self-efficacy beliefs as a proximal factor within this model may offer significant insights for explaining prospective teachers’ behavioral intentions to use new digital technologies in their practices [31]. Accordingly, the main aim of the present study is to examine a serial mediation model in which the relationship between prospective teachers’ AI literacy and their behavioral intentions to use AI in teaching is mediated by their AI self-efficacy and attitudes toward AI. Relatedly, the second aim is to adapt the Artificial Intelligence Self-Efficacy Scale (AISES) for use with Turkish prospective teachers to assess AI self-efficacy.

#### Adapting the AISES for Turkish prospective teachers

There is general agreement among researchers that AI should complement—rather than supplant—teachers, and the mere presence of advanced technologies does not automatically lead to better educational practices [4, 32]. Without their engagement and acceptance, the integration of AI into teaching practice is unlikely to succeed. However, as in many other countries, AIEd technologies have not yet been introduced into the teacher education process as part of the formal curriculum in Türkiye. Therefore, the conceptualization and measurement of psychological constructs associated with the adoption of AIEd technologies among prospective teachers, such as attitudes, anxiety, and self-efficacy, are highly relevant to current interests in educational research [33, 34]. Among these factors, AI self-efficacy beliefs may play a particularly central role, since social cognitive theory emphasizes efficacy beliefs as critical mechanisms of human agency that influence behavioral actions as well as cognitive and affective processes [35, 36]. However, to investigate AI self-efficacy beliefs among Turkish prospective teachers, a psychological assessment tool is needed, because at the time of this study, no instrument validated specifically for prospective teachers was available. Hence, this study aimed to adapt the Artificial Intelligence Self-Efficacy Scale developed by Wang and Chuang [37] into Turkish, and the subsequent hypothesis was formulated:

- H1. The Turkish version of the Artificial Intelligence Self-Efficacy Scale demonstrates adequate psychometric properties: construct validity (H1a) and reliability (H1b).

#### Testing the serial mediation model

Human thought, emotion, and behavior are shaped by the interaction of personal and environmental factors. In this interaction, an antecedent factor may relate to behavior both directly and indirectly through intervening factors [38]. These indirect associations are investigated using mediation analysis. Accordingly, the current study explores the mediation roles of prospective teachers’ AI self-efficacy and attitudes towards AI in the association between their AI literacy and behavioral intention to use AI in teaching. The theoretical background and empirical evidence supporting the serial mediation model are articulated below.

##### AI literacy as a predictor

Creating a learning environment that encourages prospective teachers to use AI applications might not be straightforward. Burgos et al. [4] highlighted two main challenges in integrating AI into the educational system, namely, how to implement it ethically and how to develop AI literacy. The growing body of research on the TAM has also identified AI literacy as a crucial determinant of the intention to use technology [39]. For instance, Cabero-Almenara et al. [40] reported that college students lacked adequate training in the effective use of AI tools and underlined the need for AI literacy initiatives. In this regard, Kajiwara and Kawabata [41] developed a curriculum to enhance AI literacy regarding large language models (LLMs). They specifically designed the program to promote the ethical use of artificial intelligence. Their study showed that the richer individuals’ knowledge of AI is, the higher the likelihood of its use. Gaining the necessary skills and education may help individuals become more familiar with the technology and more willing to adopt it in both their academic and professional environments. On the other hand, individuals who lack knowledge about AI—or worse, who harbor prejudices or perceive risks in using AI applications (e.g., misuse of personal information)—may avoid these technologies. Implementing AI literacy initiatives can help minimize perceived risks and enhance the intention to use AI applications [42]. Based on this theoretical background and previous evidence, the following hypothesis was built:

- H2. Artificial intelligence literacy predicts prospective teachers’ behavioral intentions to use AI applications.

##### AI self-efficacy and attitudes towards AI as mediators

In the social sciences, the relationship between two phenomena often operates through intervening factors known as mediators. Therefore, the present study focuses on factors that may mediate the link between literacy and behavioral intention among prospective teachers. One of the factors integrated into the TAM is perceived self-efficacy [43]. The self-efficacy theory, as posited by Bandura, pertains to individuals’ beliefs in their ability to perform a particular task effectively [44]. Individuals with a strong sense of self-efficacy maintain motivation when faced with challenges, set higher objectives, demonstrate greater commitment, and achieve higher performance [45]. On the other hand, individuals with diminished perceived self-efficacy frequently evade tasks they consider beyond their competence, struggle to cope with distress, experience anxiety, set lower objectives, and give up after minor failures [35, 46, 47]. Therefore, self-efficacy theory explicitly states that an individual’s belief in their ability to perform behaviors is a key determinant of behavioral intention and subsequent action [36].

Research indicates that computer self-efficacy is a major factor in determining teachers’ behavioral intention to use technology in educational settings [48]. Teachers who believe they can effectively use computers in classroom activities are more motivated to integrate them into real-life practice. Similarly, recent attention has turned to AI self-efficacy, defined as individuals’ perceived ability to use or operate AI applications effectively [37]. Pham and Sampson [6] noted that one factor contributing to the limited adoption of technology among teachers is their perception that they are unable to use it. Therefore, building a strong sense of AI self-efficacy among prospective teachers may be crucial for encouraging them to incorporate AI into teaching-related tasks. Moreover, developing AI literacy may contribute to higher AI self-efficacy. As outlined in the theory, gaining knowledge and skills can serve as mastery experiences that strengthen self-efficacy beliefs [44]. Grounded in this theoretical framework and prior research, the subsequent hypotheses were formulated:

- H3. Prospective teachers’ level of AI literacy predicts their AI self-efficacy.
- H4. Prospective teachers’ AI self-efficacy predicts their behavioral intentions to use AI applications.
- H5. Prospective teachers’ AI self-efficacy mediates the relation between AI literacy and behavioral intention to use AI applications.

Another critical component of TAM, bridging external factors and behavioral intention, is attitudes toward technology. One theoretical basis for hypothesized mediator role of attitudes towards AI in the present study lies in the roots of Davis’s original TAM: the theory of reasoned action (TRA). Davis [25] was inspired by the TRA, which is dedicated to explaining individual behavior in specific systems. The TRA posits that individual behaviors are determined by behavioral intention, which is controlled by attitudes and subjective norms towards the behavior [49, 50]. If individuals have a favorable attitude towards a specific behavior, they show a higher intention to perform it; vice versa. Furthermore, expectation-value theory (EVT), another theoretical concept incorporated with TAM, provides theoretical grounds for self-efficacy beliefs as an antecedent to individual attitudes. The EVT posits that attitudes are a function of one’s expectations for success and subjective value of the task [51, 52]. In simpler terms, people who believe that he/she will achieve an outcome and value the activity will have a more positive attitude towards it, have a higher behavioral intention, and eventually engage in actual behavior. According to Wigfield and Eccles [53], expectations of success align with the concept of efficacy expectations proposed by Bandura [44], an essential component of self-efficacy beliefs. Therefore, attitudes bridge self-efficacy beliefs and behavioral intention [52]. As an antecedent to self-efficacy, AI literacy might also directly predict prospective teachers’ attitudes towards AI. A recent study conducted by Yeşilyurt and Vezne [54] demonstrated that digital literacy contributed to a more positive attitude toward computer-assisted education among Turkish prospective teachers. This is particularly meaningful from the lens of the knowledge-deficit model, in which a major factor leading to unwanted behaviors or failure to engage in the behavior is a lack of knowledge [55]. Therefore, in order to cultivate positive attitudes, individuals need education and literacy. Likewise, by reading and learning about AI Technologies, prospective teachers might build a more positive attitude toward it.

- H6. Prospective teachers’ AI literacy predicts their attitudes toward AI.
- H7. Prospective teachers’ attitudes toward AI predict their behavioral intentions to use AI applications.
- H8. Prospective teachers’ attitudes toward AI serve as a mediator between AI literacy and behavioral intention to use AI applications.

In the original TAM, behavioral intention was not included; rather, attitudes served as the immediate predecessor of actual behavior [25]. The extension of TAM introduced behavioral intention as an intermediate factor between attitudes and actual behavior [43]. In this extended model, attitudes are predicted by beliefs, including self-efficacy. Based on this theoretical background and empirical findings, the present study proposes that prospective teachers’ attitudes towards AI may also mediate the relationship between their self-efficacy beliefs regarding AI (the first mediator) and behavioral intention, thereby suggesting a serial mediation mechanism. Relevant literature supports this notion, suggesting that AI literacy [56, 57] and AI self-efficacy [58, 59] can contribute to more positive attitudes. Negative attitudes toward technology are often rooted in risk perceptions, anxiety, and misconceptions that create an image of danger [60, 61]. In such cases, individuals may not trust either their ability to control the technology or the technology itself. By gaining trustworthy information, individuals can build skills, knowledge, and beliefs that ultimately foster more positive attitudes. Thus, AI literacy might strengthen teachers-in-training’s self-efficacy, grounded in enhanced abilities and knowledge, enabling them to develop more positive attitudes toward AI.

- H9. Prospective teachers’ AI self-efficacy and attitudes toward AI sequentially mediate the relationship between their AI literacy and behavioral intention to use AI applications.

## Methods

### Participants and procedure

The study sample consisted of 610 prospective teachers in total (59.5% female, *n*= 363; 40.5% male, *n*= 247) recruited via a non-proportional convenience sampling method from three state universities in Turkey. Although convenience sampling potentially holds the pitfalls of non-probability sampling methods, it allows researchers to select participants with less effort, time, and cost, and it can still provide a satisfactory sample in several situations [62]. The majority of the participants were in their first year (34.6%, *n*= 211) and second year (30.2%, *n*= 184), while 24.6% (*n*= 150) were third year, and 10.6% (*n*= 65) were fourth year. The participants’ age ranged from 18 to 40 years, with a mean of 21.74 (SD= 2.44).

The research data were gathered from the Faculties of Education at three medium-sized state universities in Turkey. These faculties include many teacher education programs in various disciplines, and the number of enrolled students ranges approximately between 1000 and 2500 in each. The research instruments were administered to participants in classroom settings through a face-to-face survey. Prior to delivering the survey packets, all potential participants were briefed by the researchers on the aims of the study, the inclusion criteria, the anonymity and confidentiality of the data collected, and their right to stop responding at any time before data collection began. The students were also told that their decision to participate would not affect their grades, relationships with their instructors, or other status, and then the survey packages were distributed to the volunteers. In addition, all participants signed a written informed consent form. Participants filled out the survey questionnaires in approximately 20 min.

### Measures

#### Artificial intelligence self-efficacy scale

This scale was developed by Wang and Chuang [37] to evaluate individuals’ self-efficacy beliefs as to the use of AI technologies and products. The scale includes 22 items and consists of four dimensions, which are factorized as assistance (AS), anthropomorphic interaction (AI), comfort with AI (CF), and technological skills (TS). Some example items are “I’m confident in my ability to learn simple programming of AI technologies/products if I were provided the necessary training” and “Some AI technologies/products make learning easier.” Participants grade each item on a scale ranging from 1 (strongly disagree) to 7 (strongly agree), and no items are reverse-coded. Overall scores on the scale range from 22 to 154, with higher scores indicating stronger self-efficacy beliefs about the use of AI. The original development study demonstrated that the four-factor AISES exhibited strong psychometric properties, including good construct, convergent, discriminant, content, and criterion-related validity. Additionally, Cronbach’s alpha coefficients were 0.958 for the entire scale and 0.942, 0.970, 0.963, and 0.869 for the subscales, respectively [37].

The current study adapted the scale into Turkish. Initially, permission to adapt the AISES was obtained from Yu-Wei Chuang, the corresponding author of the original scale. To maintain semantic and conceptual equivalence, a back-translation procedure was employed [63]. First, two independent translators translated the scale into Turkish, and then two additional linguists synthesized the two versions and back-translated the synthesis into English. An expert committee, consisting of researchers, linguists, and educational experts, reviewed all versions to finalize the Turkish version of the scale. Psychometric properties of the Turkish version of the AISES were detailed in the results section.

#### Behavioral intention to use AI in teaching

Behavioral intention to use AI in teaching was assessed using 5 items adapted from Ayanwale et al. [64]. Because the original items focused on teachers’ behavioral intentions to teach AI, they were adapted in this study to better reflect prospective teachers’ behavioral intentions to use AI technologies in their future teaching. The items used in this study are: “I will continue to learn about using AI in teaching,” “I will keep myself updated on the latest AI applications for teaching,” “I plan to spend time learning how to use AI technology in teaching in the future,” “I will pay more attention to emerging AI applications that can assist in teaching,” and “I intend to use AI to assist in my teaching.” Respondents grade each item on a scale ranging from 1 (strongly disagree) to 6 (strongly agree), resulting in an overall score range varying between 5 and 30. Higher scores mean stronger behavioral intention to use AI technologies in future teaching practices.

To evaluate the psychometric properties of the adapted scale, a confirmatory factor analysis (CFA) was performed. The CFA revealed that all factor loadings were higher than 0.40 (ranging from 0.71 to 0.92), and the scale demonstrated adequate construct validity with χ²/df= 5.40; CFI= 0.99; TLI= 0.98; RMSEA= 0.08 (90%CI [0.048, 0.128]); SRMR= 0.014. Although the RMSEA is at the limit of acceptable fit and its 90% confidence interval is relatively wide, the model could be considered acceptable based on Hu and Bentler’s [65] combination rules, since SRMR< 0.05 and TLI> 0.95. Moreover, the Average Variance Extracted (AVE) was 0.66, and the Composite Reliability (CR) was 0.91, indicating satisfactory convergent validity and construct reliability [66]. Additionally, Cronbach’s alpha coefficient was found to be 0.91.

#### Attitudes toward artificial intelligence

Attitudes toward AI were evaluated using the Positive GAAIS subscale of the General Attitudes toward Artificial Intelligence (GAAIS) scale, developed by Schepman and Rodway [67]. The Positive GAAIS subscale includes 12 items rated on a 5-point Likert scale (1= strongly disagree, 5= strongly agree). Some example items are “I am impressed by what Artificial Intelligence can do” and “Artificially intelligent systems can help people feel happier.” The GAAIS was translated into Turkish by Kaya et al. [68]. The adaptation study revealed that the Turkish version has strong psychometric properties, with Cronbach’s alpha coefficients of 0.82 for the Positive GAAIS and 0.84 for the Negative GAAIS. In this study, the Cronbach’s alpha was 0.89 for the positive GAAIS subscale.

#### Artificial intelligence literacy

The Artificial Intelligence Literacy Scale (AILS) [69] was used to assess AI literacy. It includes 12 items, grouped into four factors: Awareness, Usage, Evaluation, and Ethics. Some example items are “I can distinguish between intelligent devices and non-intelligent devices” and “I can select the most appropriate artificial intelligence for an application or product for a specific task.” Respondents rate each item on a 7-point Likert scale from 1 (strongly disagree) to 7 (strongly agree). Scores on the scale range from 12 to 84, and higher scores indicate greater AI literacy. The adaptation study demonstrated that the AILS is a valid and reliable measure of AI literacy in the Turkish population, with Cronbach’s alpha values of 0.72, 0.74, 0.76, and 0.72 for the Awareness, Usage, Evaluation, and Ethics factors, respectively [70]. The Cronbach’s alpha was 0.80 in this study.

### Data analysis

The factor structure of the Turkish version of the AISES was evaluated using confirmatory factor analysis (CFA) with robust maximum likelihood estimation. Model fit was assessed through the following indices: the ratio of the chi-square statistic to the degrees of freedom (χ²/df), Comparative Fit Index (CFI), Tucker-Lewis Index (TLI), Root Mean Square Error of Approximation (RMSEA), and Standardized Root Mean Square Residual (SRMR), with cut-off values of χ²/df< 5, CFI and TLI> 0.90, RMSEA and SRMR< 0.08 for acceptable fit; and χ²/df< 3, CFI and TLI> 0.95, RMSEA and SRMR< 0.05 for excellent fit [65, 71]. Convergent validity was evaluated with the AVE while reliability of the scale was assessed using Cronbach’s alpha, McDonald’s omega, and CR indicators. These analyses were performed using R (version 4.3.2).

After examining the psychometric properties of the Turkish AISES, the hypothesized model of the study was evaluated. First, descriptive statistics were calculated to summarize the data, and Pearson correlations were computed to examine associations among the variables. Then, to investigate whether the relation between AI literacy and behavioral intention to use AI in teaching was sequentially mediated by AI self-efficacy and attitudes toward AI, a serial mediation analysis was conducted using Model 6 in the PROCESS macro. The statistical significance of indirect effects was assessed using a bootstrapping procedure with 5000 bias-corrected samples and 95% confidence intervals. Indirect effects were considered statistically significant if corresponding confidence intervals did not include zero [72].

## Results

### Common method bias

Prior to conducting the main analysis, the research data were first checked for common method bias (CMB) because the data had been gathered using a common method at a single point in time. Harman’s single-factor test revealed that a single factor accounted for only 25.99% of the total variance, which is below the recommended threshold of 50% [73]. As such, common method bias was not a significant concern for validity of the findings.

### Psychometric properties of the Turkish AISES

**Fig. 1:**
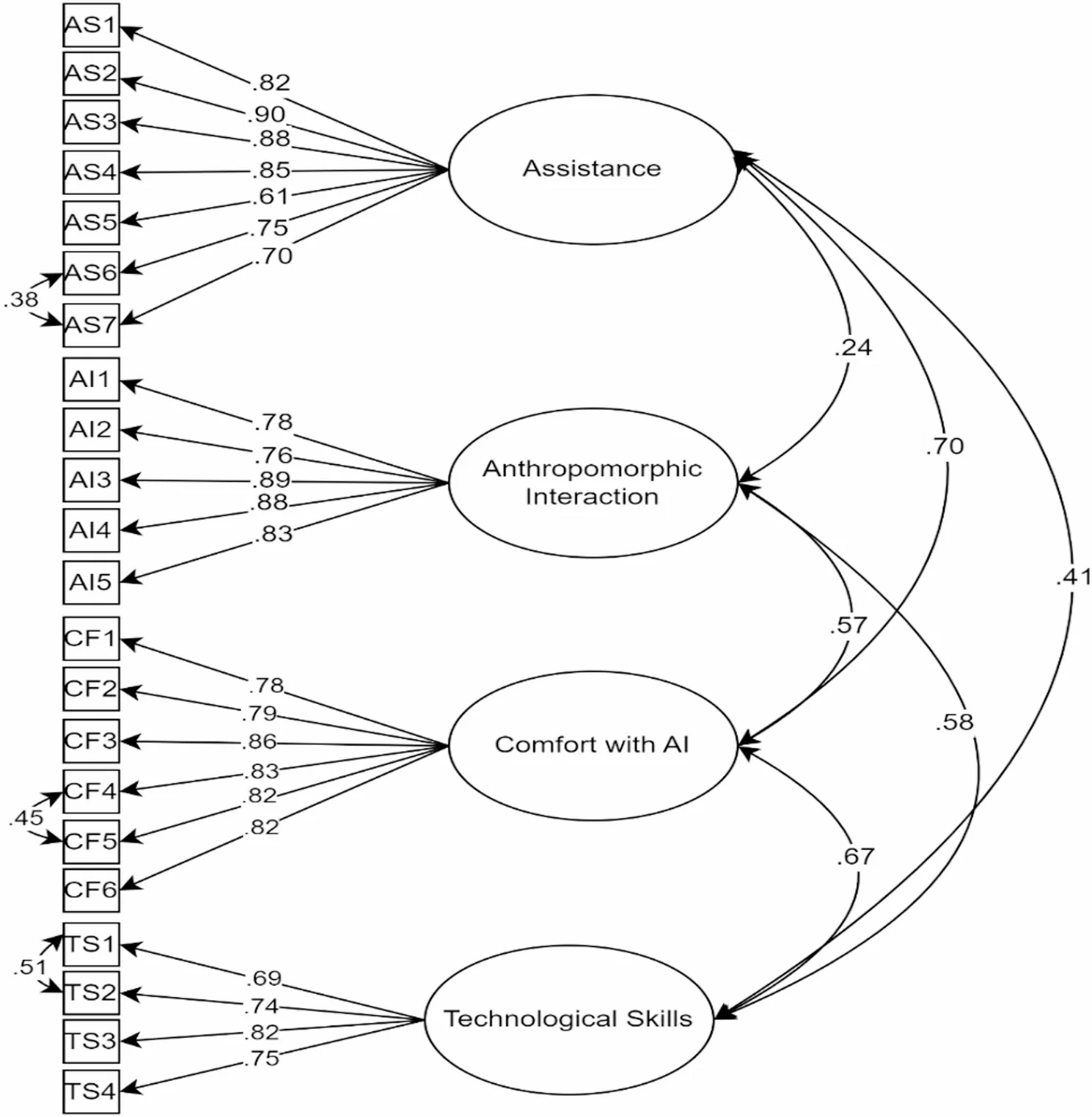
Confirmatory factor analysis results

To evaluate the construct validity of the Turkish AISES and determine whether it aligns with the four-factor structure identified in the original scale, a CFA was conducted. Initial results indicated that the hypothesized four-factor model showed a marginal fit, with *X*^2^=1088.96, *df*= 203, *p*<.05; *X*^2^/*df*= 5.36; CFI= 0.92, TLI= 0.91; RMSEA= 0.079, 90%CI [0.072, 0.085]; SRMR= 0.064. Thus, modification index was examined in order to improve model fit, and several plausible sources related to covariances between the error terms of several items indicative of misspecification were determined. High modification index values related to covariances may, apart from issues related to respondent characteristics, often arise due to semantic and conceptual overlap in item content [71]. Covariances between the measurement errors of items AS6 and AS7, CF4 and CF5, and TS1 and TS2 were therefore considered meaningful, as these pairs were located within the same sub-dimensions, indicating potential conceptual overlap. Moreover, there were logical relationships between the items in these suggested pairs, which likely resulted in shared variance between them. That is, AS6 (i.e., AI technologies/products help me to save a lot of time) and AS7 (i.e., I find it easy to get AI technologies/products to do what I want them to do) are expected to be related, because being able to obtain desired outputs from AI tools is highly likely to result in time savings for users. Similarly, the pairs CF4 (i.e., When interacting with AI technologies/products, I feel very peaceful) and CF5 (i.e., When interacting with AI technologies/products, I feel very relaxed), and TS1 (i.e., When using AI technologies/products, I am not worried that I might press the wrong button and cause risks) and TS2 (i.e., When using AI technologies/products, I am not worried that I might press the wrong button and damage it) reflect very similar emotional experiences when interacting with AI tools, indicating conceptual overlap. Consequently, these pairs of items are likely to share additional variance beyond that accounted for by the common factor. Indeed, adding covariances between error terms of these pairs resulted in a marked improvement in model fit (*X*^2^= 807.27, *df*= 200, *p*<.05; *X*^2^/*df*= 4.03; CFI= 0.95, TLI= 0.94; RMSEA= 0.063, 90%CI [0.057, 0.070]; SRMR= 0.064). Furthermore, all factor loadings in the revised model were statistically significant and exceeded the recommended threshold of 0.40, ranging from 0.61 to 0.90 (Fig. 1).

The fact that the CFA yielded satisfactory fit indices, with all standardized factor loadings exceeding the 0.40 threshold, was indicative of the Turkish version of the AISES’s convergent validity. In addition, the scale’s convergent validity was further evaluated by calculating the AVEs. The analysis showed AVE values that were higher than the recommended threshold of 0.50 [66], with values of 0.63, 0.69, 0.67, and 0.56 for the assistance, anthropomorphic interaction, comfort with AI, and technological skills subdimensions of the scale respectively, which indicated that the scale has good convergent validity.

The discriminant validity of the Turkish AISES was evaluated by the way of examining the magnitude of the correlations and Heterotrait-Monotrait (HTMT) ratios between its subdimensions. The correlation coefficients between assistance, anthropomorphic interaction, comfort with AI, and technological skills appeared to be lower than 0.75, and the HTMT ratios were below 0.85, which indicated that these subdimensions are empirically distinct constructs and supported the discriminant validity of the scale [74]). Taken together, these results supported H1a.

Cronbach’s alpha, McDonald’s omega, and CR metrics were used to assess the Turkish AISES’s reliability. CR values were 0.92, 0.91, 0.92, and 0.84 for assistance, anthropomorphic interaction, comfort with AI, and technological skills, respectively, which were higher than the recommended threshold of 0.60 [66]. Furthermore, Cronbach’s alpha for the overall scale was 0.94 and were 0.92, 0.91, 0.92, and 0.86 for subdimensions, respectively. Similarly, McDonald’s omega coefficients for these constructs were 0.94, 0.92, 0.91, 0.93, and 0.86, respectively. As such, H1b was empirically confirmed.

The psychometric equivalence of a psychological construct measured across different demographics or groups could be examined through tests of measurement invariance, so that whether that construct has the same meaning for these groups could be evaluated. As such, a measurement invariance analysis was conducted to compare the performance of the Turkish version of the AISES between males and females, and the results are summarized in Table 1. The findings from this analysis demonstrated that the differences in Satorra-Bentler scaled x^2^ across configural, metric, and scalar models were not statistically significant (*p*>.05). Besides chi-squares, differences in alternative fit indices were also evaluated. It was found that differences in CFI, TLI, and RMSEA values were lower than the recommended thresholds, i.e., ΔCFI< 0.01, ΔTLI< 0.01, and ΔRMSEA< 0.015 [75]. According to these results, the Turkish form of the AISES is able to measure artificial intelligence self-efficacy in the same way for males and females, further supporting its psychometric robustness.

**Table 1:** Measurement invariance of the Turkish AISES by gender

Invariance	X^2^	df	ΔX^2^ (df)	CFI	TLI	RMSEA	ΔCFI	ΔTLI	ΔRMSEA
Females	451.374	200							
Males	381.489	200							
Configural	832.863	400		0.940	0.931	0.071			
Metric	860.197	418	22.652(18)^ns^	0.940	0.933	0.070	0.000	0.002	− 0.001
Scalar	878.064	436	10.795(18)^ns^	0.941	0.937	0.068	0.001	0.004	− 0.002

*ns* non-significant

### Testing the hypothesized serial mediation model

Following the examination of the psychometric qualities of the Turkish AISES, the next step was to investigate the hypothesized chain mediation model. Prior to conducting the mediation analysis, descriptive statistics and Pearson correlations were computed (Table 2). AI literacy was positively correlated with AI self-efficacy (*r*= .254, *p*< .001), attitudes towards AI (*r*= .257, *p*< .001), and behavioral intention to use AI in teaching (*r*= .295, *p*< .001). Similarly, AI self-efficacy was positively correlated with attitudes towards AI (*r*= .592, *p*< .001) and behavioral intention to use AI in teaching (*r*= .562, *p*< .001). Additionally, attitudes toward AI were positively associated with behavioral intention to use AI in teaching (*r*= .534, *p*< .001).

**Table 2:** Descriptive statistics and pearson correlations

Variable	1	2	3	Mean	SD	Kurtosis	Skewness
1. AIL				60.01	9.73	1.49	− 0.616
2. AISE	0.254^***^			93.58	23.03	0.51	0.21
3. ATAI	0.257^***^	0.592^***^		43.39	7.72	1.42	− 0.57
4. BIUTA	0.295^***^	0.562^***^	0.534^***^	20.57	5.64	− 0.29	− 0.24

*AIL* AI Literacy, *AISE* AI Self-Efficacy, *ATAI* Attitudes Towards AI “positive general attitudes towards AI” subscale, *BIUTA* Behavioral Intention to Use AI in Teaching

^***^p< .001

The serial mediation model was investigated through Model 6 of the PROCESS macro in SPSS. Furthermore, age and gender were added as covariates in the model to control potential effects of these demographic characteristics. The results (Table 3) confirmed the hypothesized relationships among the study variables. Specifically, AI literacy positively predicted AI self-efficacy (B= 0.610, p< .001, 95%CI [0.429, 0.791]), which, in turn, positively predicted behavioral intention to use AI in teaching (B= 0.089, p< .001, 95%CI [0.069, 0.101]), supporting H3 and H4, respectively. Similarly, AI literacy positively predicted attitudes towards AI (B= 0.092, p<. 01, 95%CI [0.040, 0.145]), which, in turn, positively predicted behavioral intention to use AI in teaching (B= 0.213, p< .001, 95%CI [0.156, 0.270]). Therefore, H6 and H7 were confirmed. Moreover, AI self-efficacy was positively linked to attitudes to AI (B= 0.186, p <.001, 95%CI [0.164, 0.209]), leading to a serial mediation pathway. After accounting for the effects of the mediators and covariates, the direct effect of AI literacy on behavioral intention to use AI in teaching remained statistically significant (B= 0.073, p< .01, 95%CI [0.035, 0.110]), which offer support for H2. Taken together, these results suggest that AI self-efficacy and attitudes towards AI could partially mediate this relationship.

**Table 3:** Regression coefficients in the serial mediation analysis

Outcome	Predictors	*R*	*R* ^2^	F	B	SE	t	95%CI (LB, UB)
AISE	AIL	0.289	0.083	18.366	0.610	0.092	6.624^***^	(0.429, 0.791)
	Gender				6.087	1.824	3.338^**^	(2.505, 9.669)
	Age				− 0.385	0.367	-1.048^ns^	(-1.105, 0.336)
ATAI	AIL	0.604	0.364	86.849	0.092	0.026	3.471^**^	(0.040, 0.145)
	AISE				0.186	0.011	16.400^***^	(0.164, 0.209)
	Gender				0.822	0.514	1.598^ns^	(-0.188, 1.831)
	Age				− 0.026	0.103	− .252^ns^	(-0.227, 0.176)
BIUTA	AIL	0.632	0.399	80.322	0.073	0.019	3.802^**^	(0.035, 0.110)
	AISE				0.089	0.010	9.171^***^	(0.069, 0.108)
	ATAI				0.213	0.029	7.372^***^	(0.156, 0.270)
	Gender				− 0.324	0.366	− .883^ns^	(-1.043, 0.396)
	Age				0.168	0.073	2.310^*^	(0.025, 0.312)

* *p*< .05. ** *p*< .01. *** *p*< .001

**Fig. 2:**
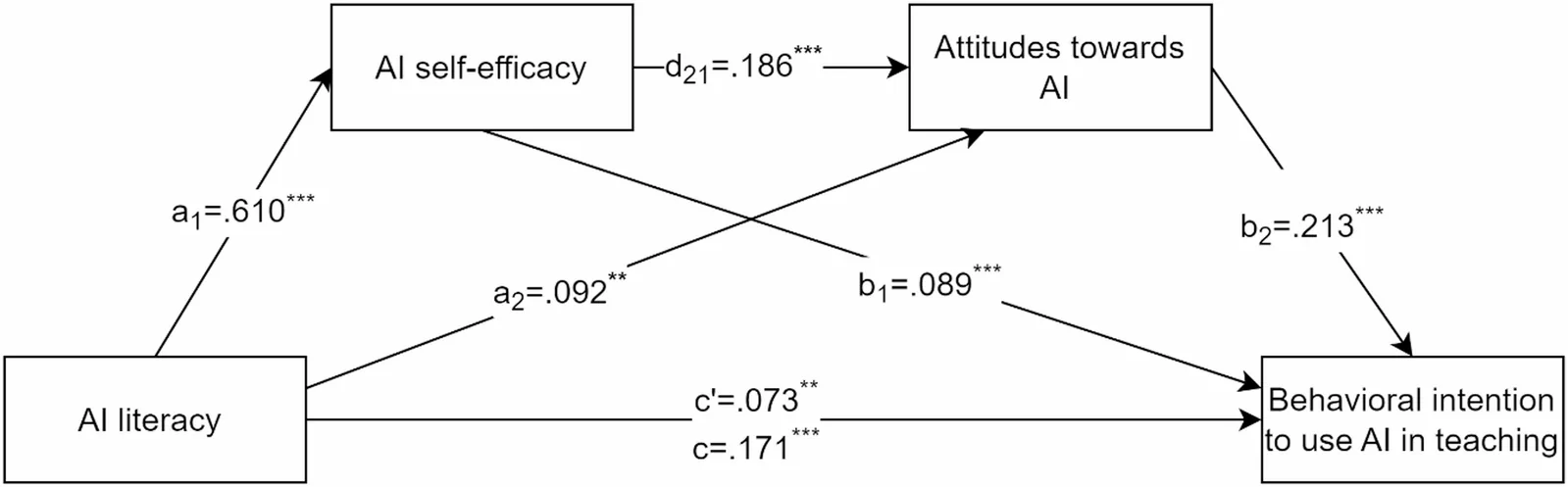
The serial mediation model

To evaluate the statistical significance of the indirect effects, a bootstrap test with 5000 bias-corrected resamples was performed. The results (Table 4) demonstrated that the total indirect effect of AI literacy on behavioral intention to use AI in teaching was statistically significant (B= 0.098, 95%CI [0.062, 0.136]), accounting for 57.31% of its total effect. There were three statistically significant indirect pathways in this association: (1) AI literacy → AI self-efficacy → behavioral intention to use AI in teaching (B= 0.054, 95%CI [0.031, 0.081]), which accounted for 31.58% of the total effect; (2) AI literacy → attitudes towards AI → behavioral intention to use AI in teaching (B= 0.020, 95%CI [0.006, 0.037]), which accounted for 11.70% of the total effect; and (3) AI literacy → AI self-efficacy → attitudes towards AI → behavioral intention to use AI in teaching (B= 0.024, 95%CI [0.012, 0.040]), which accounted for 14.03% of the total effect. Consequently, H5, H8, and H9 were supported by the bootstrapping results. Additionally, the serial mediation model (Fig. 2) explained 39.9% of the variance in behavioral intention to use AI.

**Table 4:** Direct, indirect and total effects of AI literacy on behavioral intention to use AI in teaching

Effect	B	SE/BootSE	95% CI (LB, UB)
Total effect	0.171	0.022	(0.127, 0.215)
Direct effect	0.073	0.019	(0.035, 0.110)
Total indirect effect	0.098	0.019	(0.062, 0.136)
Indirect effect 1 (AIL → AISE → BIUTA)	0.054	0.013	(0.031, 0.081)
Indirect effect 2 (AIL → ATAI → BIUTA)	0.020	0.008	(0.006, 0.037)
Indirect effect 3 (AIL → AISE → ATAI → BIUTA)	0.024	0.007	(0.012, 0.040)

## Discussion

The integration of AI tools into education encourages transformative practices in the teaching-learning process, providing substantial benefits for both students and teachers [76]. However, the effective implementation of these technologies in classroom settings primarily depends on the adoption of these tools by teachers and prospective teachers. In this context, the present study has significant implications, demonstrating that the Turkish version of the AISES can be used as a robust instrument for measuring prospective teachers’ self-efficacy in using AI technologies, and that AI self-efficacy and attitudes towards AI may serve as statistically significant mediating mechanisms that may link AI literacy to the behavioral intention to use AI in teaching among prospective teachers. However, it is important to note that these findings indicate associations rather than evidencing a causal or sequential mediating mechanism.

### Validity and reliability of the turkish version of the AISES

The first objective of this study was to adapt the AISES into Turkish and evaluate its psychometric properties among prospective teachers. As a relatively new measurement tool, there are, to the best of our knowledge, no other studies addressing its adaptation to different cultures specifially among prospective teachers. Therefore, this study significantly contributes to the existing literature by broadening the research base and providing evidence for its cross-cultural applicability and reliability.

The findings from CFA revealed that the Turkish version of the AISES adhered to the four-factor structure identified in Wang and Chuang’s [37] study, supporting the structural validity of the original scale. Additionally, the fit indices obtained in this study were similar to those of the original study, indicating a good structural validity. However, the Turkish version of the scale required a modification process of adding covariances between the items AS6 and AS7, CF4 and CF5, and TS1 and TS2 to achieve these satisfactory fit indices. Modification indices are significant statistics used to identify specific areas of misfit in a CFA and are recommended to be pursued to improve model fit if they can be justified on empirical or conceptual grounds [77]. Self-efficacy beliefs are theoretically considered as domain-specific constructs [36], and therefore, in self-efficacy scales, items that tap into the same domain of efficacy are expected to be related [78]. Given that all items with added covariance between their error terms are located within the same sub-dimensions, they can be considered thematically and conceptually related. Consequently, these modifications to the Turkish AISES can be explained on both conceptual and theoretical grounds.

The CFA results also demonstrated that, similar to its original version, the factor loadings of items in the Turkish AISES were very high, ranging from 0.61 to 0.90, 0.76 to 0.89, 0.78 to 0.86, and 0.69 to 0.82 for the subdimensions of assistance, anthropomorphic interaction, comfort with AI, and technological skills, respectively. Although there is no consensus on a minimum value for factor loadings, it is often expected to be at least 0.40 to adequately represent construct [79, 80]. Tabachnick and Fidell [81] recommend cutoff values of 0.32, 0.45, 0.55, 0.65, and 0.71 for poor, fair, good, very good, and excellent loadings, respectively. Consequently, it can be concluded that the items of the Turkish AISES are very good indicators of the constructs they are designed to measure, similar to the original scale.

As for reliability, consistent with previous findings this study achieved satisfactory results. More specifically, Wang and Chuang [37] reported Cronbach’s alpha values ranging from 0.87 to 0.95. Similarly, the alpha values in the present study ranged from 0.86 to 0.94. In addition, Omega reliability was also calculated and found to range from 0.86 to 0.94, akin to the Cronbach’s alpha values. Moreover, the fact that composite reliability values were higher than the threshold of 0.60 provided additional evidence for the reliability of the Turkish AISES. Taken together, the psychometric findings indicate that the Turkish form of the scale is a valid and reliable instrument.

### The serial mediating effect of AI self-efficacy and attitudes toward AI in the relationship between AI literacy and behavioral intention to use AI in teaching

The second objective of the present study was to explore whether AI self-efficacy and attitudes towards AI sequentially mediate the link between prospective teachers’ AI literacy and their behavioral intentions to use AI in teaching. The findings confirmed the hypothesized relationships among the variables of interest. To the best of our knowledge, this research is the first to document these specific associations, making a significant contribution to the existing body of literature. Evidence arising from this study not only underscores the role of AI literacy in the use of AI technologies in educational settings but also enriches the understanding of the mediating mechanisms involved in this process.

The findings revealed that AI literacy predicted behavioral intention to use AI in teaching, indicating that as prospective teachers’ AI literacy increases, their likelihood of adopting AI technologies in future classroom practices also rises. Previous research has identified AI literacy as a significant factor that encourages educators to adopt and integrate AI technologies into educational settings [82, 83]. Given that we live in an age of technology, AI literacy has the potential to become a skill as critical in future work and educational environments as basic literacy is today. Indeed, scholars have argued that individuals’ ability to effectively use artificial intelligence needs to be urgently developed [84]. AI literacy refers to the ability to recognize, interpret, and use AI technologies purposefully, and to evaluate the opportunities they offer [85–87]. These competencies can enable prospective teachers to develop a deep understanding of AI technologies and to enhance their ability to determine how to act effectively in the face of this rapidly evolving technology [87].

It has been observed that AI literacy serves as an antecedent in the TAM model and plays a fundamental role in shaping key outcomes, including users’ attitudes, intentions, and behaviors [39, 40]. In this context, the critical role of AI literacy in the extended TAM model theoretically supports the findings of this study. AI literacy enhances individuals’ knowledge and skills regarding this technology [41]. Increased knowledge and skills may help replace negative preconceptions about AI technologies with more realistic expectations. Put simply, individuals may hesitate to use AI due to concerns about potential risks (e.g., the malicious use of personal information). Indeed, researchers have indicated that literacy can help reduce perceived distrust, thereby fostering more positive attitudes toward AI [88].

The present study also revealed that AI self-efficacy serves as a mediating factor between AI literacy and the behavioral intention to use AI in teaching. Theoretically, AI literacy can function as a source of mastery experiences by equipping prospective teachers with the necessary knowledge and skills [44]. With greater knowledge, enhanced skills, and a stronger sense of competence, they may be more inclined to integrate AI technologies into their future classrooms. This aligns with the core assertions of self-efficacy theory. Bandura [89] emphasizes that “unless people believe they can produce desired effects by their actions, they have little incentive to act” (p. 18). Accordingly, AI self-efficacy may encourage prospective teachers to behave in ways that lead to desired instructional outcomes. Self-efficacy is also situated within the belief component of the extended TAM [43]. Moreover, previous studies have emphasized that AI self-efficacy plays a critical role in shaping both attitudes and the behavioral intention to use AI [48, 90].

The present study indicated that attitudes toward AI mediate the relationship between AI literacy and the behavioral intention to use AI. In the original TAM model, attitude was identified as a key factor influencing behavior [25]; however, in later extensions of the model, behavioral intention was positioned between attitude and behavior [43]. Within this framework, attitudes toward AI appear as a central component of the TAM. Moreover, previous research suggests that both AI literacy [56, 57] and AI self-efficacy [58, 59] play a significant role in the development of attitudes. Improved literacy is closely associated with the processes of understanding, evaluating, and effectively using the target behavior, and they often yield various benefits, such as changes in attitudes toward the domain in which literacy is acquired [91]. For instance, increased computer literacy among nurses has been shown to positively influence their attitudes toward computer-assisted medical tools [92], while medical students’ improved computer literacy has been found to foster more favorable attitudes toward e-learning [93] Therefore, AI literacy can be understood as a construct that is characterized by variables such as interest in and attitudes toward AI tools [94].

Cultivating AI literacy among prospective teachers may help them adapt to AI-assisted classrooms, feel competent in using AI tools for educational purposes, and further develop their ability to integrate these technologies. However, this remains a significant challenge, as there may still be gaps in knowledge, skills, and confidence regarding the use of even less advanced computer technologies among college students. Moreover, without proper training, AI technology can be misused by learners, leading to ethical concerns. Nevertheless, it is evident that well-designed AI literacy programs can contribute to the ethical and effective integration of AI into future education.

### Limitations, implications, and future directions

This study presents several limitations that warrant caution in interpreting the findings. First, the psychometric properties of the Turkish version of the AISES were examined using a sample solely comprised of prospective teachers. Additionally, this study could not assess test-retest reliability and cross-validation. Future research may investigate these psychometric properties using a more diverse demographic sample to enhance the scale’s generalizability and psychometric robustness. Second, the study used cross-sectional data collected at a single time point, which does not allow for the establishment of definitive causal relationships, but mediation models assume a temporal ordering between variables in their nature. Therefore, the statistical results of this study concerning the serial mediation model should be interpreted cautiously, as they reflect associations rather than indicating a sequential mediating process. Experimental or longitudinal studies could provide more robust evidence of the causal relationships suggested in this study. Third, since this study used self-reported data, the results might be affected by participant-induced errors, such as social desirability bias. Fourth, the present study focused solely on AI self-efficacy and attitudes towards AI as mediators in the relationship between AI literacy and the behavioral intention to use AI in teaching. There are likely other psychological mechanisms that could influence this relationship and are worth exploring in future studies. Fifth, the data in the present study were collected by employing a non-proportional convenience sampling method due to practical constraints, which may limit the generalizability of the findings. Future studies may benefit from employing probability-based sampling methods to obtain a more representative sample.

This study, despite its limitations, has significant theoretical and practical implications. Theoretically, the findings contribute to the adaptation and development of the TAM [25] within the AI literature by demonstrating that AI-related skills, i.e., AI literacy, can foster the use of AI products. The findings also enhance the understanding and efforts to integrate social-cognitive elements into the TAM [95], by highlighting the significant role of self-efficacy in transforming knowledge about technology into behavioral intentions to use it.

In terms of pedagogical practice, there is a growing effort and investment to develop educational AI technologies and integrate them into instructional practices with the ultimate aim of advancing teaching [96–98]. However, these investments might remain inert if they are not adopted by the end users that are teachers and students. The results of the current study suggest that AI literacy may be a critical factor in this process, as it may strengthen prospective teachers’ AI self-efficacy, foster their positive attitudes toward AI technologies, and ultimately increase their likelihood of adopting these technologies in future teaching practices. In this vein, teacher training institutions can enhance their curricula in order to include more content that may develop AI literacy both theoretically and practically. Additionally, educational training sessions and awareness studies could be conducted on fundamental themes such as the basic principles of AI technologies, their potential applications in teaching, and their pedagogical integration into the classroom.

To more effectively bridge theory and practice, AI-enabled teaching tools can be developed and integrated into teacher training programs so that prospective teachers can become more familiar with these technologies and strengthen their self-efficacy beliefs regarding their use. The study highlights that AI self-efficacy and attitudes toward AI are significant predictors of the behavioral intention to use AI technologies in teaching. Consequently, implementing and disseminating resources for developing AI skills, such as educational simulations and hands-on workshops, in teacher education can help prospective teachers build stronger self-efficacy and more positive attitudes towards AI.

## Conclusion

This study adapted the AISES into Turkish for a sample of prospective teachers, demonstrating its psychometric robustness and cross-cultural applicability. Moreover, the study documented the serial mediating roles of AI self-efficacy and attitudes toward AI in the relationship between AI literacy and behavioral intention to use AI in teaching. Therefore, this study offers valuable insights for designing interventions and educational strategies that empower prospective teachers to adopt and effectively use AI tools in their future teaching practices.

## Supplementary Information

Supplementary Material 1.

## Data Availability

The data supporting this study’s findings are available on a reasonable request from the corresponding author.
