# Supplementary material for: Turkish adaptation of the Artificial Intelligence Self-Efficacy Scale (AISES) and its role in prospective teachers’ intentions to use AI in classroom practices
Source: BMC Psychol. 2026 Jun 23;14:1284. doi: 10.1186/s40359-026-05014-2 (PMC13543356; doi:10.1186/s40359-026-05014-2)
Supplement: Supplementary file 1 [file 40359_2026_5014_MOESM1_ESM.docx]

**Supplementary Material – Turkish Version of the Artificial Intelligence Self-Efficacy Scale (AISES)**

**Yapay Zeka Öz-Yeterlik Ölçeği**

|  | **Yönerge:** Değerli katılımcı, bu ölçek yapay zekâ teknolojilerini kullanmaya yönelik düşünce ve deneyimlerinizi belirlemek amacıyla hazırlanmıştır. Lütfen her bir ifadeyi dikkatlice okuyarak sizin için en uygun seçeneği 1 ile 7 arasında işaretleyiniz. | Kesinlikle katılmıyorum | Katılmıyorum | Kısmen katılmıyorum | Kararsızım | Kısmen katılıyorum | Katılıyorum | Kesinlikle katılıyorum |
| --- | --- | --- | --- | --- | --- | --- | --- | --- |
| 1 | Bazı yapay zekâ teknolojileri/araçları öğrenmeyi daha kolay hale getirir. | 1 | 2 | 3 | 4 | 5 | 6 | 7 |
| 2 | Yapay zekâ teknolojilerinin/araçlarının öğrenme için faydalı olduğunu düşünüyorum. | 1 | 2 | 3 | 4 | 5 | 6 | 7 |
| 3 | Yapay zekâ teknolojileri/araçları öğrenme açısından iyi bir yardımcıdır. | 1 | 2 | 3 | 4 | 5 | 6 | 7 |
| 4 | Yapay zekâ teknolojilerini/araçlarını kullanmak öğrenmeyi daha ilgi çekici hale getirir. | 1 | 2 | 3 | 4 | 5 | 6 | 7 |
| 5 | Gerekli eğitimi aldığım takdirde, yapay zekâ teknolojilerini/araçlarının temel düzeyde programlanmasını öğrenme konusunda kendime güveniyorum. | 1 | 2 | 3 | 4 | 5 | 6 | 7 |
| 6 | Yapay zekâ teknolojileri/araçları sayesinde çok zaman kazanırım. | 1 | 2 | 3 | 4 | 5 | 6 | 7 |
| 7 | Yapay zekâ teknolojilerine/araçlarına yapmak istediklerimi kolaylıkla yaptırabilirim. | 1 | 2 | 3 | 4 | 5 | 6 | 7 |
| 8 | Yapay zekâ teknolojileriyle/araçlarıyla olan etkileşim sürecinin, adeta gerçek bir insanla sohbet edermişçesine oldukça canlı(gerçekçi) olduğunu düşünüyorum. | 1 | 2 | 3 | 4 | 5 | 6 | 7 |
| 9 | Etkileşim sırasında yapay zekâ teknolojilerinin/araçlarının içeriği tıpkı gerçek bir insan gibi özgün bir şekilde ifade ettiğini düşünüyorum. | 1 | 2 | 3 | 4 | 5 | 6 | 7 |
| 10 | Yapay zekâ teknolojilerinin/araçlarının diyalog biçimi ile gerçek insanlarınki arasında bir fark olmadığını düşünüyorum. | 1 | 2 | 3 | 4 | 5 | 6 | 7 |
| 11 | Yapay zekâ teknolojilerinin/araçlarının etkileşim sırasındaki üslubunun gerçek insanlarınkiyle aynı olduğunu düşünüyorum. | 1 | 2 | 3 | 4 | 5 | 6 | 7 |
| 12 | Yapay zekâ teknolojilerinin/araçlarının etkileşimli metinlerde kullandığı ifade biçiminin gerçek insanlarınki ile aynı olduğunu hissederim. | 1 | 2 | 3 | 4 | 5 | 6 | 7 |
| 13 | Yapay zekâ teknolojileriyle/araçlarıyla etkileşim kurarken kendimi çok sakin hissederim. | 1 | 2 | 3 | 4 | 5 | 6 | 7 |
| 14 | Yapay zekâ teknolojileriyle/araçlarıyla etkileşim kurmayı kolay bulurum. | 1 | 2 | 3 | 4 | 5 | 6 | 7 |
| 15 | Yapay zekâ teknolojileriyle/araçlarıyla etkileşim kurarken içim rahattır. | 1 | 2 | 3 | 4 | 5 | 6 | 7 |
| 16 | Yapay zekâ teknolojileriyle/araçlarıyla etkileşim halindeyken kendimi çok huzurlu hissediyorum. | 1 | 2 | 3 | 4 | 5 | 6 | 7 |
| 17 | Yapay zekâ teknolojileriyle/araçlarıyla etkileşim kurarken kendimi çok rahatlamış hissederim. | 1 | 2 | 3 | 4 | 5 | 6 | 7 |
| 18 | Yapay zekâ teknolojileriyle/araçlarıyla sorunsuz ve keyifli bir şekilde etkileşim kurabilirim. | 1 | 2 | 3 | 4 | 5 | 6 | 7 |
| 19 | Yapay zekâ teknolojileri/araçlarını kullanırken yanlış bir tuşa basıp sorun çıkaracağım diye endişelenmem. | 1 | 2 | 3 | 4 | 5 | 6 | 7 |
| 20 | Yapay zekâ teknolojilerini/araçlarını kullanırken yanlış bir tuşa basıp ona zarar vereceğim diye endişe duymam. | 1 | 2 | 3 | 4 | 5 | 6 | 7 |
| 21 | Bir yapay zekâ teknolojisini/aracını kullanırken nedenini anlamadığım bir durumla karşılaşmam. | 1 | 2 | 3 | 4 | 5 | 6 | 7 |
| 22 | Yapay zekâ teknolojilerinin/araçlarının jargonu(terimleri) beni şaşırtmaz. | 1 | 2 | 3 | 4 | 5 | 6 | 7 |

Alt boyutlar:

- Yardım/Destek: 1–7. maddeler
- Antropomorfik Etkileşim: 8–12. maddeler
- Yapay Zekâ ile Rahatlık: 13–18. maddeler
- Teknolojik Beceriler: 19–22. maddeler
